# Reconstructing the origin and dispersal patterns of village chickens across East Africa: insights from autosomal markers

**DOI:** 10.1111/mec.12294

**Published:** 2013-04-24

**Authors:** J M Mwacharo, K Nomura, H Hanada, J L Han, T Amano, O Hanotte

**Affiliations:** * Centre for Genetics and Genomics, School of Biology, University Park, University of NottinghamNottingham, NG7 2RD, UK; † Laboratory of Animal Genetics and Breeding, Department of Animal Sciences, Tokyo University of Agriculture1737 Funako Atsugi-Shi, Kanagawa, 243-0034, Japan; ‡ CAAS-ILRI Joint Laboratory on Livestock and Forage Genetic Resources, Institute of Animal Science, Chinese Academy of Agricultural Sciences (CAAS)Beijing, 100094, China; § International Livestock Research InstituteP.O. Box 30709, Nairobi, 00100, Kenya

**Keywords:** Bayesian inference, demographic history, *Gallus gallus*, genetic diversity, livestock, migration, trading

## Abstract

Unravelling the genetic history of any livestock species is central to understanding the origin, development and expansion of agricultural societies and economies. Domestic village chickens are widespread in Africa. Their close association with, and reliance on, humans for long-range dispersal makes the species an important biological marker in tracking cultural and trading contacts between human societies and civilizations across time. Archaezoological and linguistic evidence suggest a complex history of arrival and dispersion of the species on the continent, with mitochondrial DNA (mtDNA) D-loop analysis revealing the presence of five distinct haplogroups in East African village chickens. It supports the importance of the region in understanding the history of the species and indirectly of human interactions. Here, through a detailed analysis of 30 autosomal microsatellite markers genotyped in 657 village chickens from four East African countries (Kenya, Uganda, Ethiopia and Sudan), we identify three distinct autosomal gene pools (I, II and III). Gene pool I is predominantly found in Ethiopia and Sudan, while II and III occur in both Kenya and Uganda. A gradient of admixture for gene pools II and III between the Kenyan coast and Uganda's hinterland (*P* = 0.001) is observed, while gene pool I is clearly separated from the other two. We propose that these three gene pools represent genetic signatures of separate events in the history of the continent that relate to the arrival and dispersal of village chickens and humans across the region. Our results provide new insights on the history of chicken husbandry which has been shaped by terrestrial and maritime contacts between ancient and modern civilizations in Asia and East Africa.

## Introduction

Across Africa, domestic village chicken are raised under free-range scavenging conditions and show large variations in qualitative and quantitative traits (plumage colour, feather morphology and pattern, skin colour, comb types, live weights, egg production etc.) (Msoffe *et al*. 2001; Dana *et al*. 2010). Although chickens were domesticated in Asia (Delacour 1951; Johnsgard 1999), free-range scavenging village chickens have a long historic presence in the African continent where they sustain livelihoods for millions of people in smallholder subsistence economies. The earliest zoo-archaeological evidence for the presence of chickens in Africa trace back to ancient Egypt during the XIX Dynasty (The Ramesside period, 1307–1196 BC) (Houlihan & Goodman 1986). In the East African region, archaeological dates (calibrated) are more recent; the earliest is mid-seventeenth century BC in Sudan (Houlihan & Goodman 1986), compared to 800 AD in coastal Kenya (Marshall 2000), and in Akameru and Cyinkomane in Rwanda (MacDonald 1992; MacDonald & Edwards 1993). However, the subsequent pattern and chronology of dispersion of the species within the continent remain unclear (Mwacharo *et al*. 2013).

Domestic chicken have poor flight capability and rely entirely upon humans for medium and long-distance dispersal. Understanding their pattern of dispersal may therefore provide an indirect insight into patterns of human interaction (Mwacharo *et al*. 2013). From their geographic centres of origin and domestication in Asia, some scholars have suggested that chickens were first introduced to Africa *via* Egypt from where they dispersed southwards into East Africa following the Nile River basin (MacDonald 1992; Blench & MacDonald 2000). Other schools of thought have argued for an independent introduction directly from the Indian subcontinent and South-east Asia to East Africa *via* Indian Ocean trading networks (Chami 2001; Fuller *et al*. 2011). Indeed, the African continent and the East African region in particular have had prolonged and sustained socio-economic interactions with Asia over several thousand years. Such interactions have facilitated both maritime and terrestrial intercontinental translocations of domestic and non-domestic plant and animal species through the Horn of the continent (Beaujard 2005; Boivin & Fuller 2009; Fuller & Boivin 2009; Fuller *et al*. 2011). It would not be surprising therefore that domestic chicken would have been an intrinsic part of these interactions.

Analysis of the control region of the mitochondrial genome has led to the suggestion that domestic chicken might be derived from multiple geographic centres of origin in Asia (Liu *et al*. 2006; Miao *et al*. 2013). A recent study of East African village chickens revealed the presence of at least five mitochondrial DNA (mtDNA) D-loop haplogroups, three of which are probably derived from different ancient source populations in Asia (Mwacharo *et al*. 2011).

In Africa, village chickens have been investigated previously using autosomal microsatellite markers. In the Southern African region, a study by Muchadeyi *et al*. (2007) revealed high genetic diversity and an absence of genetic sub-structure among village chickens from Zimbabwe, Malawi and Sudan, a finding that contrasted results from mtDNA analysis which identified two distinct haplogroups that most probably were derived from the Indian subcontinent and South-east Asia (Muchadeyi *et al*. 2008). Similarly, van Marle-Koster & Nel (2000) and Mtileni *et al*. (2011) have revealed high level of genetic diversity in free-range scavenging village chickens sampled from Botswana and Mozambique and/or South Africa. More recently, Goraga *et al*. (2012) studied the genetic diversity of five Ethiopian populations (ecotypes) using 26 microsatellites and compared it with six commercial pure breeds. They found that Ethiopian village chickens are genetically distinct from commercial breeds. Interestingly, they identified two different genetic clusters amongst the Ethiopian village chickens but did not interpret further these results. In West Africa, Leroy *et al*. (2012) studied the genetic diversity, agroecological structure and introgression patterns of village chickens across North, West and Central Africa. They show evidence of gene flow between commercial and local chicken populations in Morocco and Cameroon while no clear genetic differentiation between chicken populations of Benin, Ghana and Ivory Coast was observed. However, overlaying the farming systems to their data revealed that chicken populations from the same agro-ecological zone are more related to each other compared to other populations (Leroy *et al*. 2012).

In this study, we investigated the geographic structure and genetic diversity of free-range scavenging village chickens from the East African region. We used 30 autosomal microsatellites that were genotyped in 657 individuals from 15 populations. We demonstrate that the genetic diversity found within and among the studied populations can be partitioned into three broad genetic groups with different and only partly overlapping geographic range. We further reveal that there is genetic intermixing/admixture between the three genetic groups that indicates possible ancient and/or recent migration pattern of village chickens in the region. Finally, we discuss our microsatellite results in relation to recent findings from the analysis of mtDNA, and in the light of archaeological and historical information concerning the origin, arrival and dispersal of domestic village chickens across East Africa.

## Materials and methods

### Sample collection and DNA extraction

**Fig. 1:**
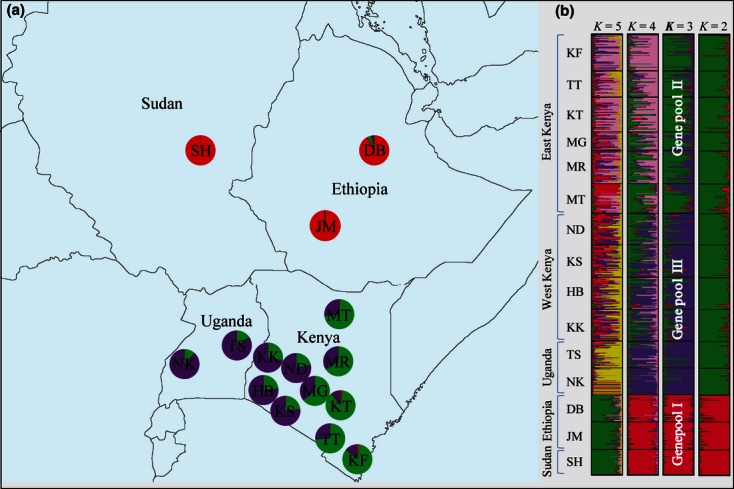
(a) Geographic distribution of village chickens. The shaded area in each pie is proportional to the number of individuals in each population observed for each gene pool. (Population abbreviations: East of Kenya: KF, Kilifi; TT, Taita; KT, Kitui; MG, Muranga; MR, Meru; MT, Marsabit; West of Kenya: KS, Kisii; ND, Nandi; HB, Homa Bay; KK, Kakamega; Ethiopia: DB, Debre Berhan; JM, Jimma; Sudan: SH, Shilluk; Uganda: TS, Teso; NK, Nkonjo). Colour codes: Red, Gene pool I; Green, Gene pool II: Purple, Gene pool III. (b) Bayesian analysis of population structure of East African village chickens. Individuals (represented by single vertical lines) are assigned to three distinct gene pools based on clustering result at *K* = 3. Colour codes: Red, Gene pool I; Green, Gene pool II; Purple, Gene pool III.

We genotyped 657 birds from 15 populations of village chickens from four countries in East Africa (Kenya = 10; Uganda = 2; Ethiopia = 2; Sudan = 1), giving an average sample size of 43 birds (range = 28–54) per population (Table 1, Fig. 1a, b). The study populations are raised under free-range backyard scavenging system and flocks from different households interact freely. Veterinary health care and nutritional management are minimal. Mating is uncontrolled, and systematic artificial selection/breeding is uncommon and natural selection for survival against diseases (Newcastle, Mareks, Salmonellosis, Infectious Bursal disease (Gumboro), pasteurella etc.), ecto- and endo-parasites and predation are the predominant selective forces. For sampling, we chose geographic locations delimited by local country specific administrative boundaries. The eco-climatic characteristics of each sampling location have been described by Pratt *et al*. (1966) and are shown in Table S1 (Supporting information). To avoid sampling genetically related birds in the absence of pedigree information, two mature birds were sampled per homestead, at a minimum sampling distance between homesteads of three kilometres. To asses possible introgression of exotic blood into indigenous chicken, four commercial lines (*n* = 112), including two layers (White Leghorns from Europe (*n* = 26) and USA (*n* = 35)), one broiler (Chunky (*n* = 35)) and one multipurpose breed (Barred Plymouth Rock (*n* = 16)) were also genotyped. These were chosen to represent the main commercial breeds usually imported into the study area for crossbreeding with local flocks to improve egg and meat production (MoALD & M 1993; Moges *et al*. 2010). Genomic DNA was recovered from blood using phenol-chloroform extraction (Sambrook & Russell 2000). The birds were genotyped for 30 autosomal microsatellite loci (Table S2, Supporting information) that have been recommended by the International Society for Animal Genetics (ISAG)/Food and Agriculture Organisation of the United Nations (FAO) Advisory Committee on measurement of domestic animal genetic diversity (FAO 1998; http://dad.fao.org/cgi-bin/getblob.cgi?sid=6e227435d25a608081d877656f3f3a32,50006220). All genotypes were double-blind scored independently by five people conversant with the scoring of microsatellites.

**Table 1:** Indicators of genetic diversity in 15 East African village chicken populations analysed using 30 microsatellite markers

		Allelic diversity					Genetic diversity		Proportion of genepools	
				
Population (code)	*N*	TNA	MNA (SD)	AR (SD)	ENA (SD)	Pa	He (SD)	Ho (SD)	I/II/III	*F*_IS_
Kenya										
Kilifi (KF)	54	196	6.53 (2.99)	5.80 (2.36)	3.12 (1.04)	5	0.65 (0.02)	0.57 (0.01)	2.1/83.9/14.0	0.12***
Taita (TT)	39	178	5.93 (2.72)	5.55 (2.38)	3.15 (1.31)	6	0.64 (0.03)	0.56 (0.01)	0.2/73.8/26.0	0.13***
Muranga (MG)	28	161	5.37 (2.58)	5.34 (2.56)	3.14 (1.43)	0	0.64 (0.03)	0.60 (0.01)	0.0/64.3/35.7	0.07**
Kitui (KT)	52	188	6.27 (2.91)	5.56 (2.41)	3.28 (1.47)	1	0.64 (0.03)	0.57 (0.01)	1.6/87.5/10.9	0.11***
Meru (MR)	50	188	6.27 (3.16)	5.70 (2.62)	3.32 (1.36)	1	0.65 (0.03)	0.58 (0.01)	0.3/65.7/34.0	0.11***
Marsabit (MT)	44	163	5.43 (2.34)	5.18 (2.10)	3.04 (1.16)	2	0.64 (0.02)	0.51 (0.01)	0.7/71.9/27.4	0.19***
East of Kenya	267	250	8.33 (4.45)	8.28 (4.41)	3.37 (1.41)	15	0.66 (0.02)	0.57 (0.01)	0.8/74.5/24.7	0.14***
Kisii (KS)	49	192	6.40 (3.28)	5.80 (2.72)	3.31 (1.60)	0	0.65 (0.02)	0.59 (0.01)	0.7/22.5/76.8	0.10***
Nandi (ND)	47	184	6.13 (3.20)	5.66 (2.68)	3.38 (1.42)	2	0.66 (0.02)	0.59 (0.01)	0.1/21.5/78.4	0.10***
Homa Bay (HB)	47	185	6.17 (2.95)	5.60 (2.50)	3.20 (1.46)	2	0.64 (0.02)	0.58 (0.01)	0.1/23.2/76.7	0.10***
Kakamega (KK)	48	203	6.77 (3.47)	6.07 (2.83)	3.44 (1.50)	4	0.67 (0.03)	0.61 (0.01)	0.1/28.1/71.8	0.08***
West of Kenya	191	238	7.93 (4.45)	7.91 (4.43)	3.41 (1.57)	8	0.66 (0.02)	0.59 (0.01)	0.3/23.8/75.9	0.10***
Overall	458	265	8.83 (4.95)	8.80 (4.91)	3.43 (1.49)	23	0.66 (0.02)	0.58 (0.01)	0.5/49.2/50.3	0.13***
Ethiopia										
Debre Berhan (DB)	40	158	5.27 (2.26)	4.95 (2.00)	2.67 (1.49)	2	0.55 (0.03)	0.49 (0.01)	91.9/4.9/3.2	0.11***
Jimma (JM)	42	156	5.20 (2.50)	4.82 (2.15)	2.52 (1.37)	3	0.54 (0.03)	0.45 (0.01)	99.9/0.0/0.1	0.17***
Overall	82	181	6.03 (2.94)	5.99 (2.91)	2.74 (1.65)	5	0.56 (0.03)	0.47 (0.01)	95.9/2.4/1.7	0.16***
Uganda										
Teso (TS)	40	167	5.57 (2.56)	5.23 (2.27)	2.79 (1.22)	5	0.59 (0.03)	0.53 (0.01)	0.1/15.5/84.4	0.10***
Nkonjo (NK)	40	153	5.10 (2.17)	4.87 (1.99)	2.76 (1.21)	3	0.59 (0.02)	0.51 (0.01)	0.0/11.8/88.2	0.13***
Overall	80	188	6.27 (2.90)	6.23 (2.88)	2.83 (1.24)	8	0.59 (0.03)	0.52 (0.01)	0.0/13.7/86.3	0.13***
Sudan										
Shilluk (SH)	37	144	4.80 (2.19)	4.55 (1.94)	2.31 (0.93)	6	0.51 (0.03)	0.46 (0.01)	99.9/0.1/0.0	0.11***
Non-Kenyan Overall	199	229	7.63 (4.03)	7.61 (4.02)	3.05 (1.58)	19	0.61 (0.02)	0.49 (0.01)	65.3/5.4/29.3	0.21***
Across East Africa	657	285	9.50 (5.56)	5.90 (2.60)	3.40 (1.59)	42	0.66 (0.02)	0.55 (0.01)	32.9/27.3/39.8	0.12***

N, Sample size; TNA, Total number of alleles; MNA, Mean number of alleles; AR, Allelic richness; ENA, Effective number of alleles; Pa, Private alleles detected in a single population; He, Expected heterozygosity; Ho, Observed heterozygosity; SD, Standard deviation; *F*_IS_, Coefficient of inbreeding (significant values are as indicated ***P < 0.001; **P < 0.01).

### Microsatellite amplification and genotyping

Polymerase chain reaction (PCR) products were obtained in 10 μL multiplexed reactions containing 10 ng template DNA, 1X Buffer (Promega), 10 pm of each primer, 2.5 mm of each dNTP, 1.5 mm MgCl_2_ and 1 unit of *Taq* DNA polymerase (Promega). All amplifications were carried out on an Applied Biosystems 9700 Cetus thermal cycler and involved an initial denaturation at 95 °C (5 min), 35 cycles of denaturation at 95 °C (1 min), primer annealing at temperatures varying between 58 and 62 °C (1 min) and extension at 68 °C (1 min). A final extension step at 72 °C (1 min) completed the PCRs. Genotyping was carried out on an ABI PRISM 3100 automated capillary sequencer using the GS400HD Rox internal lane size standard. Allele size calling and binning were carried out with GeneMapper v3.5 using the 3rd Order Least Squares regression method (Applied Biosystems). All loci were examined for technical artefacts with the software Micro-Checker (van Oosterhout *et al*. 2004).

### Data analysis

Allelic diversity (total number of alleles, mean number of alleles (MNA), allelic richness, polymorphic information content (PIC), effective number of alleles) and genetic diversity (expected (*He*) and observed (*Ho*) heterozygosity) were estimated from allele frequencies with FSTAT 2.9.3.2 (Goudet 2001) and Microsatellite toolkit (Park 2001) and POPGENE 1.32 (Yeh *et al*. 1997). Total genetic variation of the populations (*F*_IT_) was partitioned into within (*F*_IS_) and among population (*F*_ST_) components following Weir & Cockerham (1984). For each locus-population combination for the global data set and population groupings, we used Fisher's exact test with Bonferroni correction to test possible deviations from Hardy–Weinberg equilibrium (HWE) using GENEPOP 3.4 (Raymond & Rousset 1995). Exact *P*-values were estimated using the Markov chain algorithm with 10 000 dememorizations, 500 batches and 5000 iterations per batch.

We used Bayesian clustering algorithm implemented in STRUCTURE 2.3.3 (Pritchard *et al*. 2000; Falush *et al*. 2003) to infer population structure and explore the assignment of individuals and populations to specific genetic clusters. For this analysis, we allowed the number of clusters (*K*) to vary between 1 ≤ *K* ≤ 15, using a burn-in of 50 000 followed by 100 000 Markov Chain Monte Carlo (MCMC) iterations. Ten simulations were carried out for each *K* assuming four scenarios: (i) populations are admixed and allele frequencies correlated; (ii) populations are admixed and allele frequencies independent; (iii) populations are not admixed but allele frequencies are correlated; and (iv) populations are not admixed and allele frequencies are independent. To estimate the most optimal *K*, we used three approaches. First, we used the best log-likelihood score resulting in the highest percentage of membership coefficient (*q*) to each cluster (Pritchard *et al*. 2000). Second, the number of clusters (*K*) was plotted against *ΔK* = *m*|*L*”(*K*)|/*s*|*L*(*K*)| and the optimal number of clusters identified by the largest change in log-likelihood (*L*(*K*)) values between the estimated number of clusters (Evanno *et al*. 2005). Third, we adopted Pritchard *et al*. (2000) suggestion that for real-world data in which identifying the correct *K* is not always straightforward; the best choice of *K* should be the one that reveals a biologically meaningful genetic structure. DISTRUCT (Rosenberg 2004) was used to generate a graphical display of the simulated results.

To further generate additional information to assist in interpreting the results from STRUCTURE and therefore, correctly infer the underlying genetic structure, we used the Factorial Correspondence Analysis (FCA) implemented in GENETIX 4.05 (Belkhir *et al*. 1999) and the Principal Coordinate Analysis (PCA) implemented in ADE4 package (Dray & Dufour 2007) in the R-environment (R Development Core Team 2006). FCA portrays the relationship between individuals or populations based on the detection of the best linear combination of allele frequencies. PCA, on the other hand, clusters individuals using proportionate data based on allele frequency information. By comparing the clustering solutions generated by STRUCTURE, FCA and PCA, we defined clusters of village chickens for subsequent population genetic analyses.

The possible influence of single loci on the observed genetic structure revealed by STRUCTURE, FCA and PCA was assessed using the Multiple Co-inertia Analysis (MCoA) (Chessel & Hanafi 1996) implemented in ADE4 package (Dray & Dufour 2007) in the R-environment (R Development Core Team 2006). MCoA reveals common features of single marker analyses, generates a reference structure and makes it possible to compare population structures from single-markers with the consensus reference structure generated from the simultaneous analysis of all the markers. Using the MCoA, we estimated typological values (*Tv*) for each marker; the contribution of markers to the construction of the reference typology, which is equal to the product of the variance (Var) multiplied by the congruence with the consensus Cos^2^ (i.e. the correlation between the scores of individual locus tables and the synthetic variable of the same rank) (Laloë *et al*. 2007).

Demographic history of the populations was investigated by assessing whether or not East African village chicken populations are at mutation-drift equilibrium (MDE). We searched for signals of population expansions or contractions using four statistical approaches. Using the program Bottleneck (Cornuet & Luikart 1997), we first carried out the *T2*-test with the modified two-phase mutation model (TPM) (Garza & Williamson 2001) of microsatellite evolution and second, the qualitative descriptor of allele frequency distribution (mode shift indicator) test. The former (*T2*-test), detects recent bottlenecks on the principle that a reduction in effective population size leads to an exponential decay in heterozygosity and allele numbers at polymorphic loci and that reduction in allelic diversity is more pronounced and faster than the decline in heterozygosity (Cornuet & Luikart 1997). The latter (mode shift indicator test) reveals a bottleneck at some point in the history of a population, if a deviation from the L-shaped allele frequency distribution is observed. The parameters for the TPM were set such that 88% of the mutations followed the stepwise mutation model and 12% followed a multistep one with a variance of nine (Di Rienzo *et al*. 1994). Significant departures from MDE, within and across populations were tested using the one-tailed Wilcoxon test. Third, we used the intra-locus kurtosis test (*k*-test) and the inter-locus variance test (*g*-test) (Reich & Goldstein 1998; Reich *et al*. 1999) for MDE. The *k*-test is based on the understanding that allele distribution patterns in expanding populations differ from those that are demographically stable. In expanding populations, the kurtosis (*k*), or the combination of the variance and kurtosis (Reich *et al*. 1999), of the allele size distributions is positive. The method uses a binomial test of the number of positive *k*-values based on the expectation of an almost equal probability (*P* = 0.515) of negative and positive *k*-values. The *g*-test, on the other hand, compares the observed and estimated values of the variance in allele sizes across loci. In stable populations, the variance is highly variable among loci, whereas in expanding populations, it is much more even. For this test, low variances in allele sizes may be taken as evidence of expansion, and we used the cut-off values given in Table 1 (page 455) of Reich *et al*. (1999) for inference purposes. Both the *k*- and *g*-tests were performed using the Macro program ‘kgtests’ (Bilgin 2007) implemented in Microsoft Excel®.

As a livestock species closely associated with human activities and societies, the genetic structure of domestic chicken may be influenced by genetic improvement through crossbreeding with commercial stocks, past migration and geographic dispersion patterns. To investigate whether any of the genetic clusters revealed by STRUCTURE, FCA and PCA were influenced by introgressions from commercial breeds, the 112 individuals from four commercial breeds we genotyped were included in a separate STRUCTURE analysis with all the indigenous birds. The parameters and settings used previously to investigate the genetic structure of the indigenous fowls were employed in this STRUCTURE analysis. Individual village chickens with a membership coefficient (*q*-value) of above 0.2 for the commercial cluster were regarded to be influenced by commercial breeds. The possibilities of non-random associations between genetic differentiation, measured as [*F*_ST_/(1-*F*_ST_)] (Rousset 1997), and geographic distances, in kilometres, were tested using the IBDWS 3.05 (http://ibdws.sdsu.edu). Geographic distances between populations were calculated using the MapCrow Travel Distance Calculator (http://www.mapcrow.info/) as the distance between the central most towns within each sampling locations. To investigate the ecological specificity of any genetic clusters generated by STRUCTURE, FCA and PCA as an indirect indicator of adaptation to different ecological zones (eco-zones), we tested whether any of the genetic clusters were associated with any of the eco-zones (Table S1, Supporting information) spanning the study area. For this test, we evaluated the magnitude and significance of correlations between the genetic clusters and eco-zones using Kendall's *tau* and Spearman's *rho* statistics.

Locus *F*_ST_ values across populations were used to test the hypothesis of diversifying selection acting at each locus. We used here two approaches, the FDIST2 outlier test (Beaumont & Nichols 1996) implemented in LOSITAN (Antao *et al*. 2008) and the Bayesian approach implemented in BayeScan (Foll & Gaggiotti 2008). We chose these two methods because they have the lowest type I and II error rates (Narum & Hess 2011). For FDIST2, we carried out 100 000 simulations with a cut-off probability value of 0.99. For BayeScan, we set a value of 10 as the prior odds for the neutral model with a false discovery rate (FDR) of 0.05 and retained 550 000 iterations of the (MCMC) simulations to ensure convergence of the posterior distributions with minimal MCMC chain autocorrelation. We focussed on outlier loci suggested to be under diversifying (positive) selection only, although the two methods can also detect outlier loci showing significantly low *F*_ST_ values indicating balancing selection. Indeed, microsatellite loci characterized by high mutation rates may show significantly low *F*_ST_ outlier values independent of any balancing selection pressures (Beaumont 2008). The analysis was performed for each cluster generated by STRUCTURE, FCA and PCA.

## Results

After genotyping quality controls, which involved double-blind scoring by five people, and independent re-genotyping of loci with alleles that differed by a single base pair, 285 alleles were observed at the 30 autosomal microsatellite loci across 657 individuals from 15 village chicken populations. These included alleles differing by an uneven number of base pairs at 14 loci (MCW0216, MCW0014, MCW0183, ADL0278, MCW0104, MCW0069, MCW0034, LEI0234, MCW0016, MCW0037, LEI0094, MCW0284, LEI0192 and MCW0081). Using MICROCHECKER, we did not detect any consistent evidence for occurrence of null alleles across populations. Further details regarding the microsatellite loci and their allele sizes are presented in Supplementary information S1.

The MNA was 9.50 ± 5.56 and the effective number of alleles was 3.40 ± 1.59 across village chicken populations (Table 1). The Teso population from Uganda and Shilluk population from Sudan had, respectively, the highest (5) and lowest (0) number of loci deviating from HWE after Bonferroni correction and no single locus deviated consistently from HWE in all populations. Non-Kenyan chickens were significantly (*P* < 0.001; Wilcoxon rank-sum test corrected for multiple comparisons) less variable (MNA = 7.63 ± 4.03; AR (Allelic richness) = 7.61 ± 4.02; *He* = 0.61 ± 0.02) than Kenyan ones (MNA = 8.83 ± 4.95; AR = 8.80 ± 4.91; *He* =0.66 ± 0.02) (Table 1). Overall, chickens from Ethiopia and Sudan had the lowest genetic diversity. Comparison of the diversity between chicken populations within Kenya revealed that those from the Eastern side of the country (KF, TT, MG, KT, MR, MT) had higher allelic diversity (MNA = 8.33 ± 4.45; AR = 8.28 ± 4.41) than those from the Western side (KS, ND, HB, KK) (MNA = 7.93 ± 4.45; AR = 7.91 ± 4.43) (Table 1). However, these estimates of allelic and genetic diversity between these two groups did not differ significantly (*P* > 0.05), although a marked difference between *Ho* and *He* can be observed in all the populations analysed.

STRUCTURE, FCA and PCA analyses reveal the genetic partitioning of the observed microsatellite diversity at the individual and population level. We ran STRUCTURE with *K* varying from 1 to 15 assuming different models of population admixture and allelic correlations (see materials and methods). All scenarios tested gave similar results (Fig. 1b; Fig. S1, Supporting information). A graphic display of the estimated membership coefficients of each individual to each cluster at 1 ≤ *K* ≤ 5 assuming no admixture between populations and correlated allele frequencies is shown in Fig. 1b. To select the most suitable *K*, the best log-likelihood score approach ‘Ln*P*(*D*)’ was first applied but no distinct plateau of the estimates of the Ln*P*(*D*) was observed (Fig. S2a, Supporting information). We then plotted the *ad hoc* statistic *ΔK* (rate of change in the log probability of data between successive *K*-values) against *K* (Fig. S2b, Supporting information). The curve reveals an upper *ΔK* value for two groups (one structure covariate) indicating two distinct clusters. These two clusters group together Sudan and Ethiopia chicken on one hand and Kenya and Uganda chicken on the other. However, Fig. 1b and Fig. S1 (Supporting information) reveal that at *K* = 3, there is a further separation of the populations, which decay when *K* is increased beyond four clusters for the Kenyan and Ugandan chickens but the cluster incorporating Ethiopian and Sudanese chickens remains distinct. We further performed another STRUCTURE run using only Kenyan and Ugandan chickens. As expected, the delta *K* approach revealed an upper *ΔK* value of two groups lending further support to the existence of two genetic groups in Kenyan and Ugandan chicken as seen in Fig. 1b; one group puts together populations from East Kenya (KF, TT, MG, KT, MR, MT) on one hand and those from West Kenya (KS, ND, HB, KK) and Uganda on the other.

**Fig. 2:**
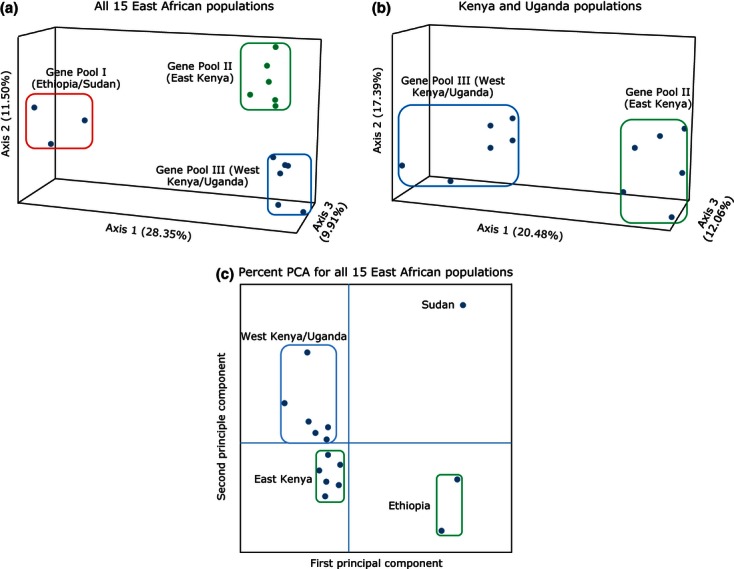
Clustering patterns of 15 populations of indigenous chicken from four countries in East Africa analysed using 30 microsatellite markers. (a) The clustering pattern of 15 indigenous chicken populations revealed by Factorial Correspondence Analysis (FCA). (b) The clustering pattern of indigenous chickens from Kenya and Uganda only revealed by FCA. (c) The clustering pattern of 15 indigenous chicken populations revealed by Principal Coordinate Analysis.

FCA and PCA clustered the study populations into three distinct groups with a clear separation of Ethiopian and Sudanese populations from Kenyan and Ugandan ones. Kenyan populations were further split into two groups, the first one included populations from the Eastern part of Kenya, while the second had populations from the Western part of Kenya including the two populations from Uganda (Fig. 2a–c). The clustering pattern of populations on the FCA and PCA plots correspond to the three distinct genetic clusters observed in STRUCTURE at *K* = 3 (Fig. 1b).

Taking into account all these results (STRUCTURE, FCA and PCA), and our previous knowledge on the mtDNA D-loop haplogroup pattern in these populations (Mwacharo *et al*. 2011), we chose *K* = 3 as the most optimal number of genetic clusters for the data set. For brevity, we refer to these three clusters as gene pools I (red), II (green) and III (purple) (Table 1; Fig.1a, b).

The three gene pools occur in the four countries in different proportions. In Ethiopia, gene pool I occurs with the highest frequency (95.9%), while the other two are observed at very low frequencies (II at 1.65% and III at 2.45%), and in only four birds which show a mixed genetic ancestry of the three gene pools. Gene pools I (99.9%) and III (0.1%) are found in Sudan, with III occurring in a single bird of mixed genotype. All three gene pools are observed in Kenya and Uganda where gene pools II and III are the most frequent. Interestingly, gene pool II occurs with a high frequency in populations found in the Eastern side of Kenya, while gene pool III is predominantly found in populations from the Western side of Kenya and in Uganda. Gene pool I, which is mainly found in Ethiopia and Sudan, occurs in Kenya and Uganda with a maximum population frequency of 0.59% and 0.05%, respectively, and in individuals showing a mixed genetic make-up between the three gene pools. The spatial geographic distribution of Gene pool II and III overlap to reveal an East–West genetic cline, II decreases in frequency from the Eastern side of Kenya to the hinterland of Uganda and the opposite is observed for III (Fig. 1a, b).

Table 1 shows the proportion of membership in each gene pool for the 15 populations. We detected admixture, defined here as individuals with less than 90% of the proportion of a single gene pool, in 235 individuals (Kenya = 209; Uganda = 19; Ethiopia = 6; Sudan = 1). The highest levels of admixture, predominantly of two gene pools (II and III), are observed in Kenya and Uganda while populations from Ethiopia and Sudan are genetically homogeneous with nearly exclusively a single gene pool (I) across all individuals. Some individuals (58 of 657) show a mixed genetic makeup between the three gene pools (I, II, III). Such admixture may explain the high levels of genetic diversity observed in chickens from Kenya and Uganda compared to those from Ethiopia and Sudan (Table 1). Forty-two population-specific alleles were observed, 23 in Kenyan and 19 in non-Kenyan chicken populations (Table 1).

To further understand the genetic differentiation between gene pools, we excluded the 235 admixed individuals from the data set, leaving a total of 423, and reanalysed the data. We observe 274 alleles out of which 162 (59.12%) are now shared between the three gene pools (Table S3, Supporting information). Gene pools I and II share 13 alleles, I and III share seven, and II and III share 24. On the other hand, we observe 66 gene pool-specific alleles ranging in frequency from 0.003165 (across eight alleles from 4 loci) to 0.085526 (one allele at one locus) (Tables S4a, b, Supporting information); 13 in gene pool I, 26 in II and 27 in III. It does not only indicate that gene pools II and III differ from gene pool I, but also reveals not only a relatively high level of admixture between gene pools II and III but possibly also a common ancestry. Re-running STUCTURE after removing admixed individuals reveals again the presence of the three gene pools (Fig. S3a, Supporting information).

Private rare alleles can in some cases influence the observed genetic pattern. We therefore reanalysed the data after removing all the individuals with such alleles. We also went further and excluded all the loci showing alleles differing by one base pair and those having irregular microsatellite allele distribution patterns (i.e. used only loci showing typical microsatellite pattern of either di- or tri-nucleotide variation) and re-ran STRUCTURE. In all cases, we ended up observing the three gene pools in the study populations with identical geographic distribution and similar pattern of genetic diversity (Fig. S3b; Table S5, Supporting information).

Figure S4a and S4b (Supporting information) shows the variance (Var), similarity (Cos2) and typological (Cov2) values for each marker for the 1st and 2nd principal components of the reference structure (Fig. 2c) generated using the MCoA. Cov2 reflects the ability of a marker to display the reference structure. The higher it is, the better the marker displays the reference structure. Cos2 on the other hand gives an indication of the congruence across markers for the global picture (Berthouly *et al*. 2008; Laloë *et al*. 2007). Of the 30 loci, six (MCW0216, MCW0037, MCW0069, MCW0067, LEI0192, MCW0330) showed Cos2 and Cov2 values of greater than 4% for the first principal component (Fig. S4a, Supporting information) which separated gene pool II from III. Similarly, ten markers (ADL0268, MCW0016, LEI0094, LEI0234, MCW0183, MCW0111, MCW0069, MCW0206, ADL0278, MCW0330) showed Cos2 and Cov2 values greater than 4% for the second principal component (Fig. S4b, Supporting information) which separated gene pool I from II and III. These results suggest that the global structure (Fig 2c) reflects the structure issued from these six and ten markers for the first and second principal components respectively. To confirm this finding, we re-performed the MCoA first excluding the six and then the 10 markers from the analysis. The analysis returned the same result. Gene pool II was still separated from gene pool III on the first principal component (Fig. S4c, Supporting information). Similarly the second analysis that excluded ten markers returned the same result for the second principal component (Fig. S4d, Supporting information) which still clearly separated gene pool I from gene pools II and III. These results suggest that each marker contributes, although at different levels, to the distinctiveness of the three gene pools (Fig. 2c).

The global estimate of *F*_ST_ indicated a low but significant level of population structuring (*P* < 0.05) between the 15 village chicken populations (Table 2). Removing the admixed individuals from the data set increased the global *F*_ST_ value from 0.05 ± 0.005 to 0.076 ± 0.007. A hierarchical analysis of *F*_IT_ revealed that heterozygote deficiency was highest across the populations of Sudan, Uganda and Ethiopia, but lowest among Kenyan populations. The inbreeding coefficients (*F*_IS_) for each population and across groups of populations (Table 1) were positive and significant (0.001 ≤ *P* ≤ 0.05) indicating a deficiency in heterozygotes. Excluding one individual from each homestead and recalculating the *F*_IS_ values returned similar results indicating that this was not an effect of the sampling strategy but possibly of long-term mating between closely related birds and/or small effective population sizes. Overall, low *F*_ST_ and high *F*_IS_ values were observed, and it is most likely that the effects of inbreeding are counterbalanced by genetic intermixing due to extensive exchange and movement of genetic stocks among local farmers.

**Table 2:** Estimates of global *F*-statistics for various groups of village chickens sampled from East Africa derived using 30 microsatellite loci

Population	*F*_IT_ ± SE	*F*_ST_ ± SE	*F*_IS_ ± SE
Kenyan village chickens (10 populations)	0.13 ± 0.02	0.019 ± 0.02	0.11 ± 0.021
Kenya and Uganda (12 populations)	0.14 ± 0.02	0.024 ± 0.002	0.11 ± 0.020
Kenya, Ethiopia and Sudan 13 populations)	0.16 ± 0.02	0.051 ± 0.005	0.12 ± 0.019
Uganda, Ethiopia and Sudan (5 populations)	0.23 ± 0.02	0.11 ± 0.013	0.13 ± 0.017
All East African village chickens (15 populations)	0.16 ± 0.02	0.05 ± 0.005	0.12 ± 0.018

Figure S5a (Supporting information) shows a graphic display of the estimated membership coefficients of each individual to each cluster at 1 ≤ *K* ≤ 5 when commercial breeds are included in the analysis and assuming no admixture between populations and no correlation among allele frequencies. Although Evanno *et al*. (2005) approach shows that the most optimal number of cluster is at *K* = 2, there is a spike at *K* = 5 indicating a further substructure in the data set (Fig. S5b, Supporting information). At *K* = 5, the three gene pools revealed previously in village chickens are observed including another two that are specific to commercial breeds and no village chicken has a membership coefficient of above 0.2 for the commercial cluster. However, performing the analysis assuming admixture and correlation among allele frequencies reveals four chicken out of 267 included in gene pool II as well as six birds out of 191 included in gene pool III with individual membership coefficients of above 0.2 for the cluster of commercial birds (range: 0.208–0349). It excludes the possibility of any gene pool to be significantly influenced by commercial introgression.

Regression analysis between pairwise geographic distances (expressed in km) from the Kenyan coastal town of Mombasa against the proportions of gene pools II and III across Kenyan and Ugandan populations reveals a significant negative regression coefficient (*b* = −0.869; *R*^2^ = 0.630; *P* = 0.001) between geographic distance and the proportion of gene pool II and a significant but positive one (*b* = 8.587; *R*^2^ = 0.634; *P* = 0.001) between geographic distance and the proportion of gene pool III (Fig. S6, Supporting information). Moreover, Mantel test, performed by regressing [*F*_ST_/(1−*F*_ST_)] (Rousset 1997) with pairwise geographic distances (km) between populations, indicated that the pattern of isolation-by-distance has been preserved among East Kenyan and West Kenyan and Ugandan village chickens (*b* = 0.04957; *P* < 0.0001). This therefore means that there has not been enough time for population movements to obscure the genetic signatures of the initial colonization of the region by the three distinct gene pools.

Association between ecological zones and the spatial distribution of the three gene pools returned significantly negative correlations (Kendall's *tau* = −0.511, calculated *P* = 0.034; Spearman's *rho* = −0.566, calculated *P* = 0.028) indicating no relationship between the eco-zones and the three gene pools. Indeed, the distribution of the three gene pools cut across different ecological zones. For instance, all three gene pools are found in eco-zone II and gene pool I occurs in eco-zones II, III and V and across different countries (Table S1, Supporting information).

To assess whether the three gene pools reflect genetic signatures of local adaptation, FDIST2 and BayeScan analyses were performed. Indeed, if local adaptive divergence gave rise to the three gene pools, some of the microsatellites might be under selection through hitchhiking linkage disequilibrium of these expected neutral markers with selected chromosomal regions (Andolfatto 2001). Using both approaches, no locus was flagged out as being under positive selection in each of the three gene pools (Fig. S7, Supporting information).

Four statistical tests were employed to detect demographic increases and/or declines in population sizes. When we analysed each population separately using the *T2*-test, we observed no significant heterozygote deficiency (0.065 ≤ *P* ≤ 0.755) or excess (0.251 ≤*P* ≤ 0.978). However, when all the 15 populations were pooled together, the *T2*-test revealed significant heterozygote deficiency (*P* = 0.001). We also found a heterozygote deficiency (0.001 ≤ *P* ≤ 0.046) when the three gene pools were analysed individually or when taking into account all the three gene pools together (*P* = 0.004). On the other hand, the mode shift indicator test did not detect any shift in allele frequency distribution across populations, at individual population levels, across gene pools and for individual gene pools. Both the intra (*k*)- and inter (*g*)- locus tests revealed evidence of demographic expansions across populations and across the three gene pools. Across populations, the *k*-test revealed 9 of 30 loci to have a positive kurtosis, a number which was significantly different from the expected binomial distribution (*P* = 0.014). Across gene pools, 10 of 30 loci showed a positive kurtosis, a number that differed significantly from the expected binomial distribution (*P* = 0.035). Similarly, six, ten and nine loci showed a positive kurtosis for gene pools I, II and III respectively, and these were also significantly different from the expected binomial distribution (*P* ≤ 0.05). The *g*-test revealed a *g*-ratio of 4.826 across populations, 7.095 across the three gene pools, 7.005 for gene pool I, 7.201 for gene pool II and 7.672 for gene pool III. From Table 1 of Reich *et al*. (1999), for sample sizes ≥160 and for 30 loci, a *g*-ratio >0.32 at *P* < 0.05 is sufficient to reject the null hypothesis of MDE.

Last but not least our results indicate a positive correlation between the proportions of autosomal gene pools and mtDNA haplogroups in the same populations (Mwacharo *et al*. 2011). More particularly between gene pool II and mitochondrial haplogroup A (Spearman's *rho* = 0.695, *P* = 0.026), and between gene pool III and mitochondrial haplogroup D (Spearman's *rho* = 0.790, *P* = 0.006) in Kenya and Uganda (see Table 3; Fig. S8, Supporting information).

**Table 3:** Spearman's correlation coefficients (*ρ*) between mitochondrial DNA (mtDNA) haplogroups (A and D)* and microsatellite gene pools (II and III) found in Kenya and Uganda

	mtDNA haplogroups	
	
Microsatellite gene pools	A	D
II	0.695; *P* = 0.026	−0.721; *P* = 0.019
III	−0.702; *P* = 0.024	0.790; *P* = 0.006

* From Mwacharo *et al*. (2011).

## Discussion

In this study, we use microsatellite markers to reveal the geographic distribution pattern of within- and between-population autosomal genetic diversity among village chickens from East Africa. For the 15 village chicken populations studied here, their heterozygosity values exceeded those estimated for highly inbred lines (Zhou & Lamont 1999) and for some local European and Asian breeds (Berthouly *et al*. 2008). Nevertheless, the mean values are similar to those estimated for some local Asian, African and Latin America populations (Wimmers *et al*. 2000; Muchadeyi *et al*. 2007). The high within-population genetic diversity observed in this study is congruent with the high variability observed in phenotypic traits in the populations and is characteristic of large traditional livestock populations that have not been under strong artificial selection pressure (Lauvergne *et al*. 2000). However, our microsatellite analysis revealed a marked difference in *Ho* and *He* in all populations (Table 1). A common assumption for most free-range scavenging village chickens is that they are panmictic. The expectation, therefore, is that *Ho* and *He* would not differ significantly. However, in the study populations, *Ho* was much lower than *He*. The possible presence of inbreeding as supported by an *F*_IS_ value > zero in each population (Table 1) could explain such result. Alternatively, it might be a consequence of the sampling strategy (see Materials and methods), which may have resulted in pooling discrete non-interbreeding subpopulations with different allele frequencies into a single randomly mating unit (Wahlund effect).

We observed three gene pools (I, II and III) across village chicken populations within the East African region (Table 1; Fig. 1a, b). Our genetic analysis support separate arrivals of the three pools rather than signals of divergence between gene pools following a single arrival in the region. Indeed, we find no support for the effect of selection and demographic history on the geographic distribution pattern of genetic diversity. Also, we find no evidence that the presence of any of the three gene pools is influenced by adaptation to eco-climatic characteristics of the region and even introgression from exotic commercial stocks. However, it has not escaped our mind that selection may be acting at other loci not investigated in this study and that such selection pressure may be too weak to be detected by our experimental design. Detailed admixture analyses further support a separate origin and routes of introduction for the three gene pools. Gene pool I is observed mainly in Ethiopia amongst our northernmost studied populations. While a high level of admixture is observed between gene pools II and III, it is not the case between I and II as well as between I and III (Table 1). So gene pool I has remained relatively isolated from the other two. Detailed allelic analyses also reveal that gene pool I shares much fewer alleles with gene pools II and III than the latter two (Table S3, Supporting information). The most likely scenario is therefore that the presence of gene pool I is the result of an independent arrival of chicken in the study area, which subsequently has remained relatively isolated from surrounding populations. The two tests of bottleneck gave conflicting results. In any case, if a bottleneck has occurred in the populations, it is likely to have been triggered by cyclic outbreaks of diseases such as Newcastle, Mareks, Gumboro etc. that are known to inflict heavy mortalities in village flocks across Africa (Gueye 1997) rather than a founding effect of the gene pools in the region. The decline in population sizes after such disease epidemics are normally followed by recoveries which may explain the signals of expansion that are detected by the *k*- and *g*-tests.

Gene pools II and III are largely admixed with significant East–West or *vice versa* gradients of admixture observed (Fig. 1a, b) while gene pool I remains relatively isolated. These two gene pools might have been introduced together and the today observed pattern of admixture might be the result of genetic drift following chicken dispersion or the two gene pools might have a separate African history. We believe that the former scenario is unlikely. If the two gene pools had been introduced simultaneously, we would have expected that populations at the entry point would show the largest diversity (e.g. Hanotte *et al*. 2002). This is not the case, with, for example, coastal populations not being more diverse than the interior ones. Also, a single introduction of the two gene pools would have led to, more or less, an even proportion of the two pools within populations across birds. Again, this is not the case (Fig. 1a, b). While, we do observe a predominant gene pool in each population, we also do find in the same populations birds belonging to each gene pool, probably a legacy of past and/or recent movements. So our data rather suggest a distinct introduction of gene pool II and III with the geographic frequency distribution pattern of gene pool II supporting its coastal arrival followed by East–West dispersion [Fig. 1a, b; Fig. S6 (Supporting information)].

Interestingly, other recent molecular studies have revealed the presence of several gene pools in the region. Mwacharo *et al*. (2011) have characterized the control region of the mitochondrial genome of 344 out of the 657 birds studied here. They reveal the presence of five distinct haplogroups in East Africa. Excluding the two rare ones of likely commercial origin, one common one (haplogroup D) was observed across the four countries, one commonly in Kenya (haplogroup A) and the third (haplogroup E) was observed at lower frequencies, in Sudan and Ethiopia. Also Goraga *et al*. (2012), who used 26 of the 30 microsatellite markers used in this study, identified two genetically distinct groups of Ethiopian village chicken populations.

Could any of the autosomal gene pools observed amongst East African village chicken be the result of recent introgression of commercial chicken lines? Recently, Leroy *et al*. (2012) have revealed gene flow between commercial and local chicken populations in Morocco and Cameroon. In our study, we included four reference breeds of commercial chicken in a separate STRUCTURE analysis. Although exotic commercial breeds have been introduced in the study area for crossbreeding purposes (MoALD & M 1993; Moges *et al*. 2010), we observed only 10 of 657 individuals with *q*-values >0.2 for the clusters of commercial breeds [Fig. S5a, b (Supporting information), *K* = 5] indicating that introgression of commercial blood into indigenous flocks may be negligible or limited and such introgression has not had any major impacts on the population structure. It does not therefore explain the existence of the three gene pools in the region. In their D-loop study, Mwacharo *et al*. (2011) identified only two birds showing haplotypes possibly of commercial origin. Based on these two findings, we hypothesize that the genetic influence of commercial breeds might still be too minor to be revealed.

It is worth mentioning that linguistic and archaeological evidences are supporting the presence of more than one gene pool of domestic chicken on the African continent. More particularly, linguistic evidences suggests at least three separate introductions of domestic chickens to West Africa; two across Central Africa from the East coast of Africa and one from the North across the Sahara (Williamson 2000). Archaeological data place the presence of domestic chickens in Egypt and inland Sudan much earlier than in any East African coastal region including inland Kenya or Uganda (Coltherd 1966; Houlihan & Goodman 1986; MacDonald 1992; Marshall 2000), while the history of agriculture in the Horn of Africa strongly supports direct movements of crops and livestock between Ethiopia, the Arabian Peninsula and the Indian subcontinent (Boivin & Fuller 2009; Fuller & Boivin 2009; Fuller *et al*. 2011), as well as between coastal East Africa and South-east Asian Islands (Beaujard 2007; Blench 2008, 2010). More particularly, it has been shown that the Horn of Africa was an entry point for at least two other livestock species, zebu cattle (*Bos indicus*) and fat-tailed sheep (*Ovis aries*) (Hanotte *et al*. 2002; Muigai & Hanotte 2013). Archaeological and historic evidences are also indicating that the East African region has witnessed major human population interactions and movements (Newman 1995; Ehret 2002) which have contributed to the dispersion of livestock within and beyond this area. For example, it is well documented that the region was a secondary centre of dispersion of Bantu speaking communities which subsequently may have played a role in the dispersion of cattle towards the southern part of the continent (Hanotte *et al*. 2002). On the other hand, it is also worth remembering that the Ethiopian highlands had remained relatively isolated for several centuries following the expansion of Islam from the eighteenth to nineteenth century AD onwards (Ehret 2002). Such events may have impacted the geographic pattern of the chicken genetic structure revealed here.

In the absence of comparable detailed microsatellite information across the geographic range of domestic chicken in Asia, we can only speculate about the Asiatic origins of the autosomal gene pools I, II and III, their entry points in the Horn of Africa and subsequent dispersion. In this respect, it is worth mentioning that we do observe a positive correlation between the proportions of autosomal gene pools and mtDNA haplogroups in the same populations (Mwacharo *et al*. 2011) [see Table 3; Fig. S8 (Supporting information)]. An arrival along the East Coast of Africa, possibly from South-east Asia and linked to the Austronesian expansion has been proposed to explain the presence of haplogroup A in Africa (Muchadeyi *et al*. 2008; Razafindraibe *et al*. 2008), while haplogroup D appears to be found commonly on the Indian subcontinent (Liu *et al*. 2006; Kanginakudru *et al*. 2008), making these two geographic regions possible centres of origins for the autosomal gene pools. Also, these results would support a coastal arrival of gene pool II and subsequent dispersion inland. As for gene pool III, its presence in Western Kenya and Uganda would be compatible with either an arrival along the Nile valley following an early introduction of domestic chicken in Egypt (MacDonald 1992; Blench & MacDonald 2000), or it could have originated from West Africa, following the arrival of Bantu speaking communities in today Uganda and around the Lake Victoria area (Newman 1995). While, these two scenarios remain possible to explain the presence of gene pool I, its origin might also be the result of an early adoption of domestic chicken by the Ethiopian agricultural societies (Fuller & Boivin 2009; Fuller *et al*. 2011). Gene pool I would then represent the legacy of ancient trading contacts between the ancient Ethiopian civilization, the Arabian Peninsula and the Indian subcontinent. Again, analysis of more populations within the continent and reference populations from outside the continent may further clarify these possible scenarios.

In conclusion, our study provides the first detailed empirical assessment of the spatial distribution of genetic diversity and structure of indigenous village chickens in East Africa. The results indicate that there is moderate-to-high genetic diversity among East African village chickens that can be attributed to three distinct gene pools. The spatial distribution of the three gene pools independent of any eco-climatic influence, selection pressure and/or introgression of exotic commercial blood is compatible with the arrival of three independent waves of domestic chicken in Africa. Our results underline the complex history of the species in the Horn and the East African region and illustrate the important role of the later for understanding the agricultural history of the continent. Our findings also support an intricate web of interactions between Asia and the East African region along maritime and terrestrial corridors but also within the Horn of Africa. Last but not least, along with other studies (Hanotte *et al*. 2002; Kijas *et al*. 2009; Larson *et al*. 2012; Warmuth *et al*. 2012), our findings further demonstrate the importance of bi-parental transmitted genetic markers in revealing in great detail the history of domestic livestock.

## Data accessibility

Microsatellite genotypes: DRYAD doi:10.5061/dryad.28fb0.

## Supporting information

**Table S1** Characteristics of the ecological zones of the study area and the respective gene pools observed in each eco-zone.

**Table S2** Characteristics of the thirty ISAG/FAO recommended microsatellite markers used in the current study.

**Table S3** Number of alleles shared between the three gene pools observed in the current study.

**Table S4a** Total number of gene pool specific alleles observed from the analysis of thirty microsatellite markers in 15 chicken populations.

**Table S4b** Gene pool specific alleles and their frequencies.

**Table S5** Measures of allelic and genetic diversity for each population after excluding individuals with less than 90% of their genotype proportion from each gene pool.

**Fig. S1** Bayesian analysis of population structure in East African village chickens based on 30 microsatellite loci for three of the four models (1, 2 and 4, see materials and methods) tested in the current study.

**Fig. S2** Estimated number of populations (K) from the program STRUCTURE for East African village chickens.

**Fig. S3** Bayesian analysis of population structure in East African village chickens.

**Fig. S4** Distribution across loci of variance (Var), similarity (Cos2) and typological (Cov2) values for each marker for the components of the first and second axes in percentages (a and b).

**Fig. S5 a)** Bayesian analysis of population structure in East African village chickens after including four breeds of commercial chickens.

**Fig. S6** Regression analysis between geographic distance (Km) from Mombasa at the Kenya Coast to the hinterland of Uganda with the proportion (%) of gene pools II and III in the two countries.

**Fig. S7** *F*_ST_ values from 30 microsatellite loci for the three gene pools observed in East Africa plotted against (a) expected heterozygosity as obtained from Lositan and (b) against log10(q-value) generated using BayeScan.

**Fig. S8** Spatial geographic distribution pattern of mtDNA haplogroups and the three microsatellite gene pools observed among village chickens from the East African region
