# Supplementary material for: Reconstructing the origin and dispersal patterns of village chickens across East Africa: insights from autosomal markers
Source: Mol Ecol. 2013 Apr 24;22(10):2683–97. doi: 10.1111/mec.12294 (PMC3664419; doi:10.1111/mec.12294)
Supplement: Supplementary file 1 [file mec0022-2683-SD1.pdf]

Supplementary Figure S1

Model 1: Populations are admixed and their allele frequencies are correlated

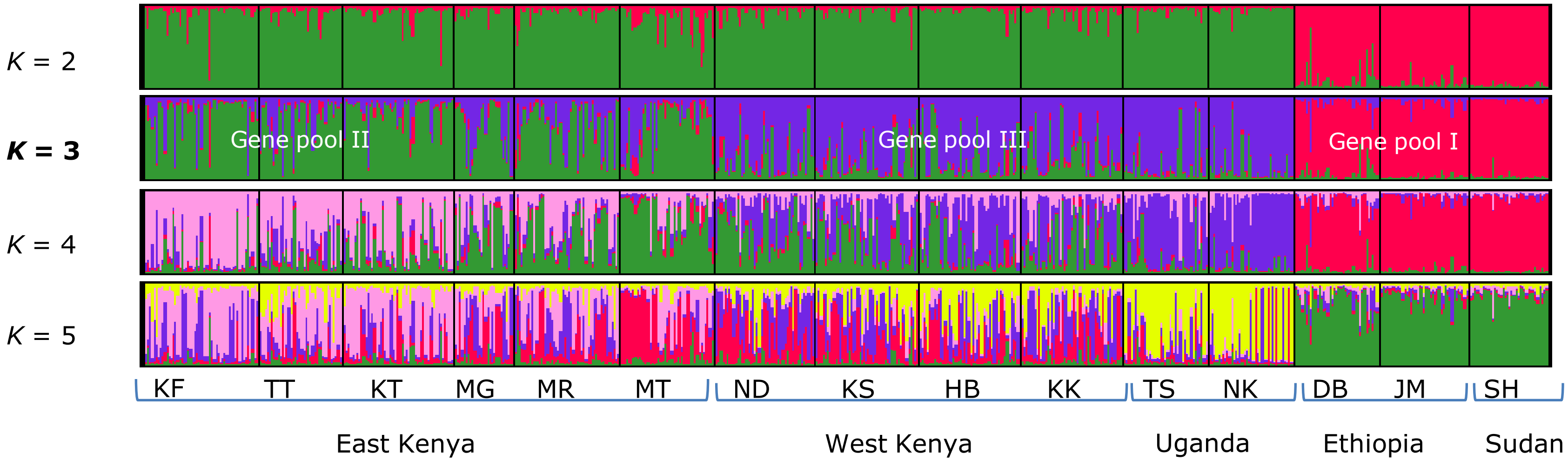

Model 2: Populations are admixed but their allele frequencies are independent

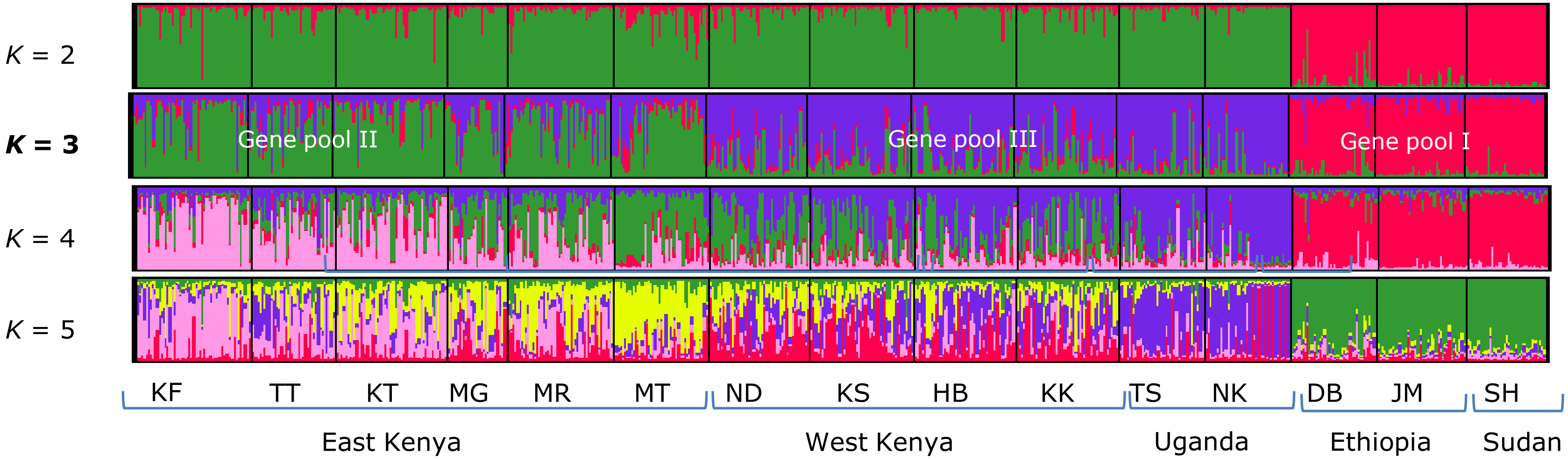

Supplementary Figure S1 continued

Model 4: No admixture between populations and their allele frequencies are correlated

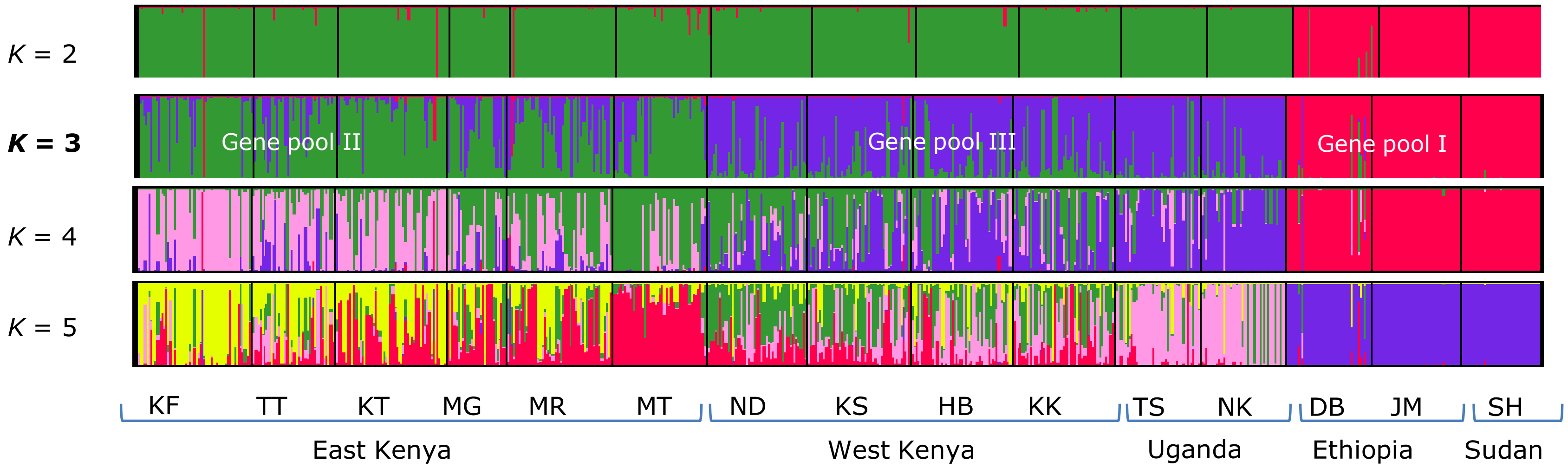

Supplementary Figure S2

A

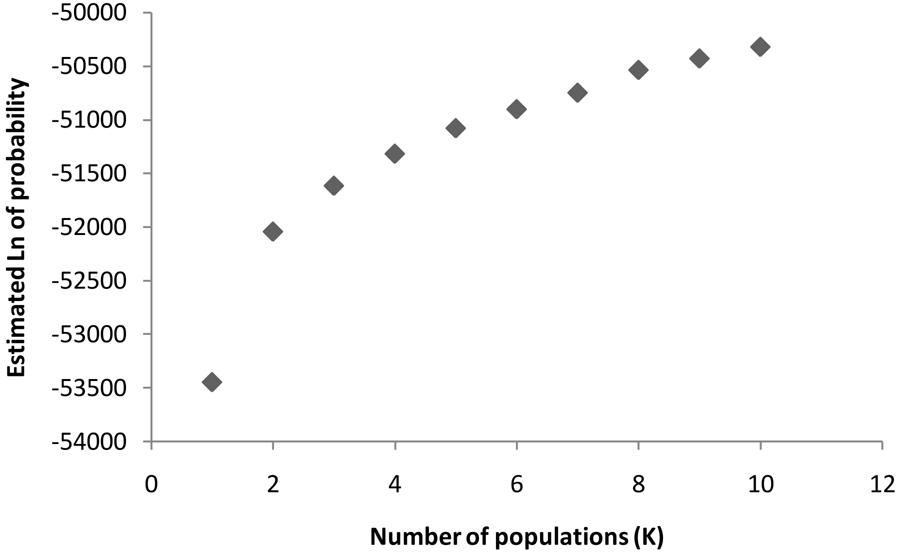

B

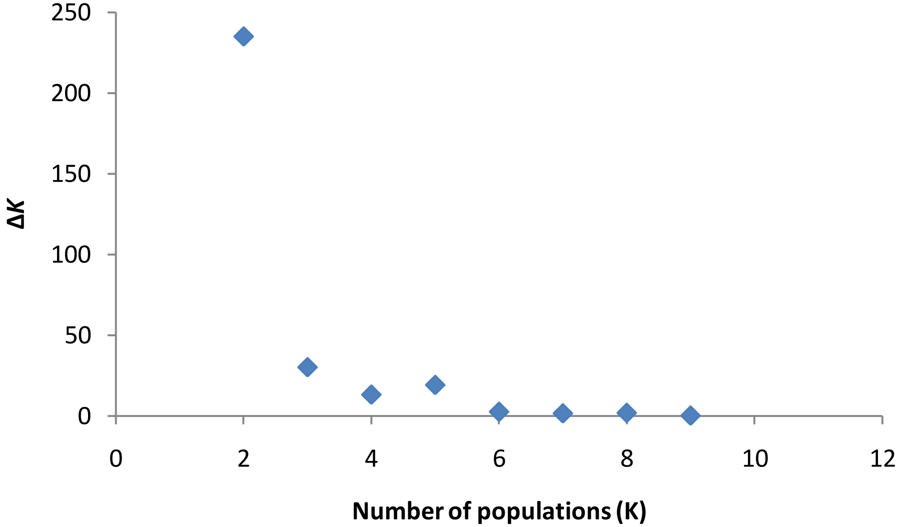

Supplementary Figure S3

Supplementary Figure S3a

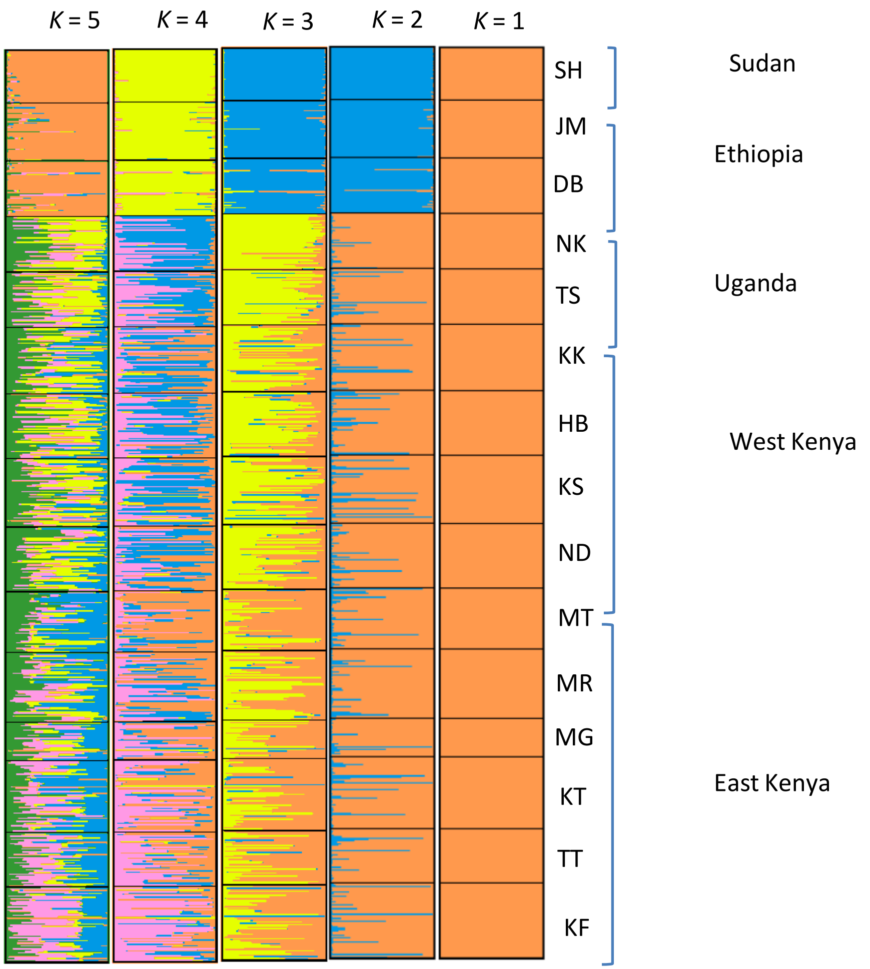

Supplementary Figure S3b

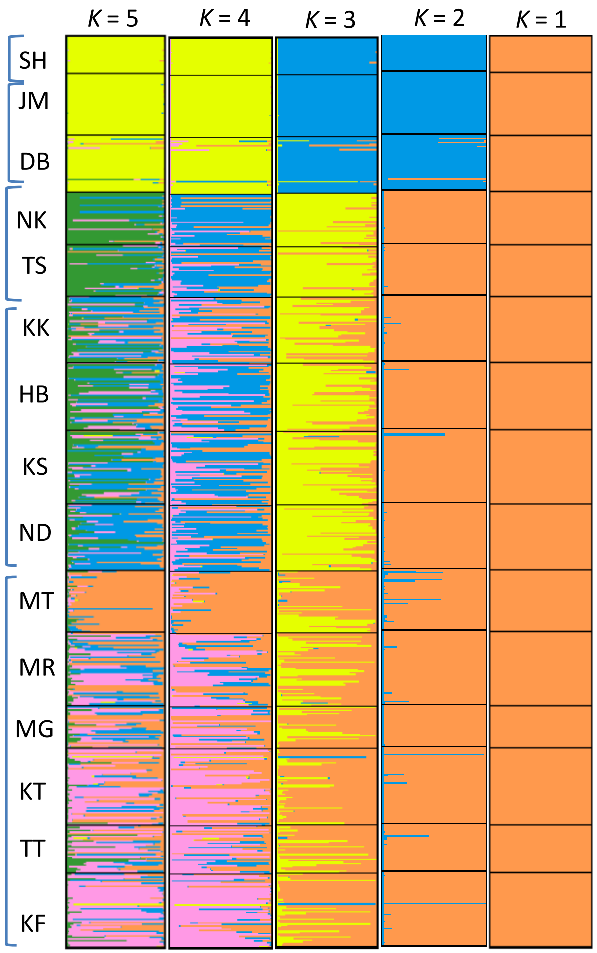

Supplementary Figure S4

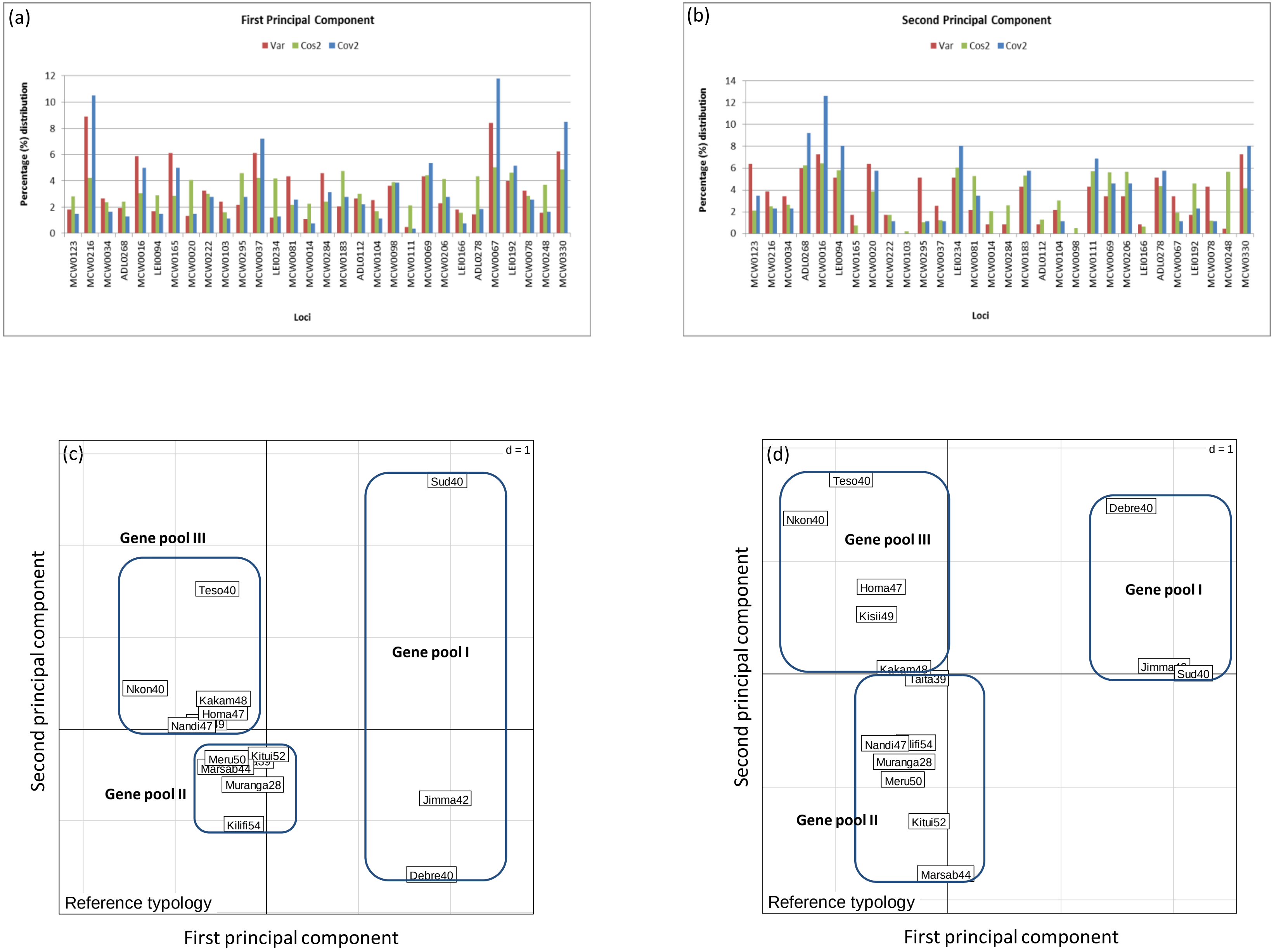

Supplementary Figure S5

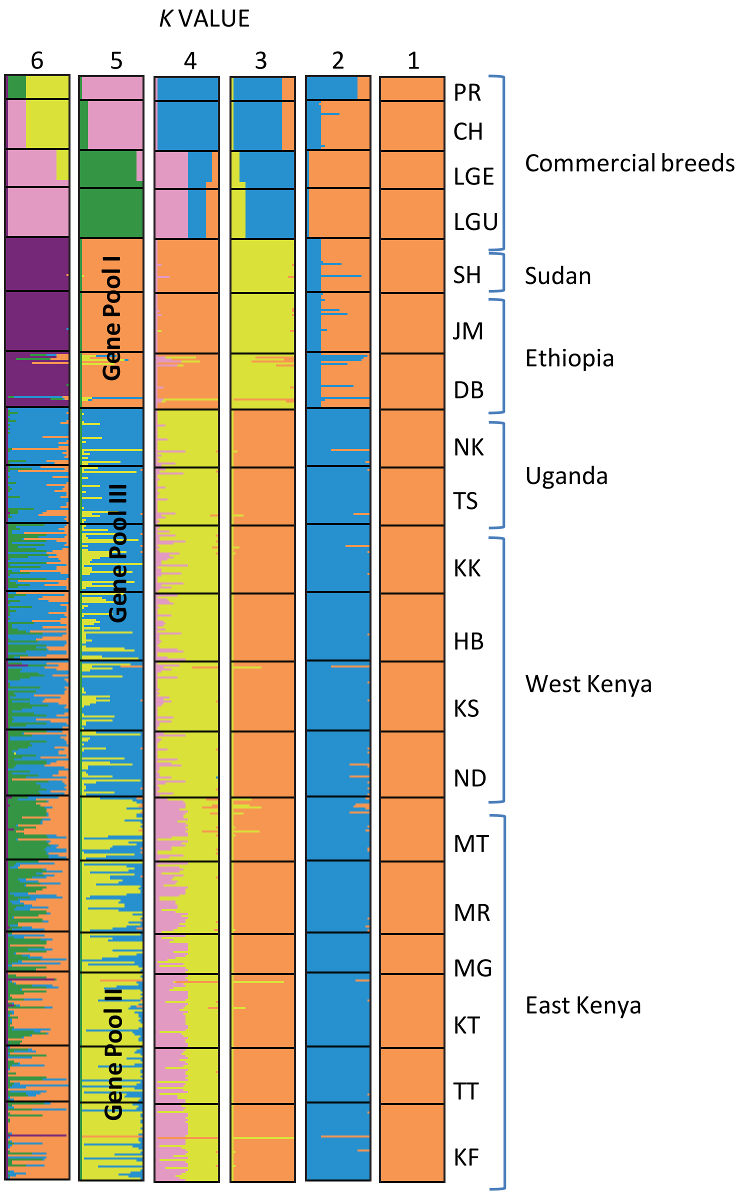

Figure S5a

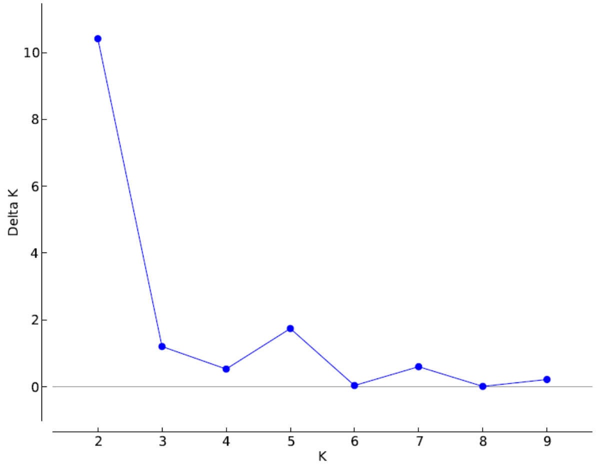

Figure S5b

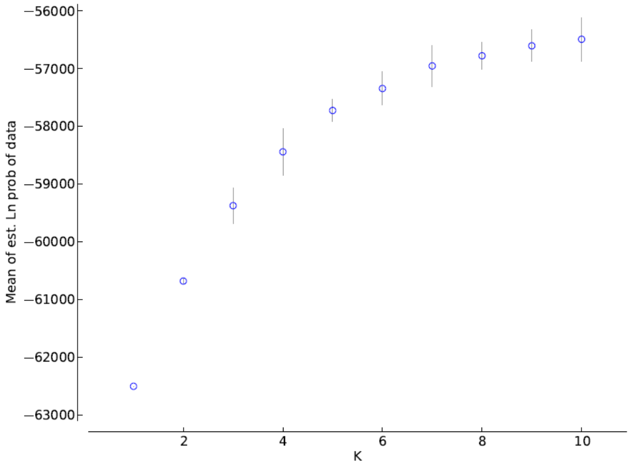

Figure S5c

Supplementary Figure S6

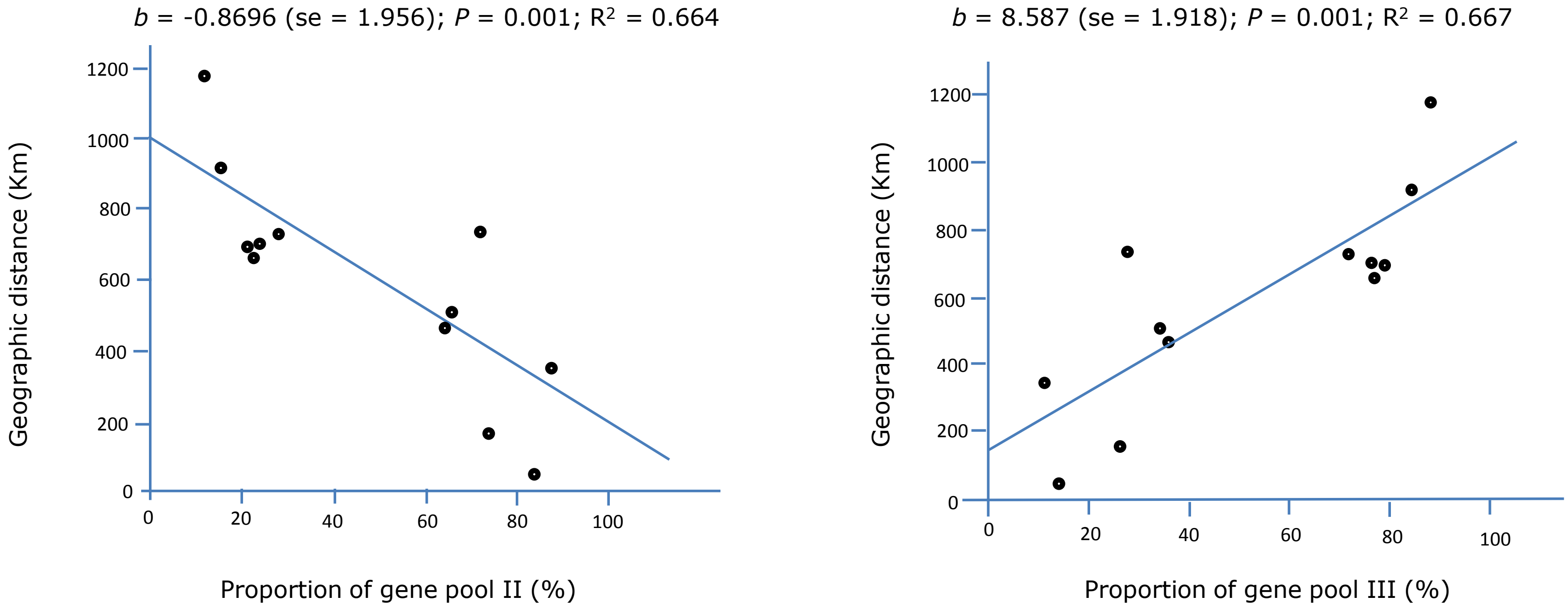

Supplementary Figure S7

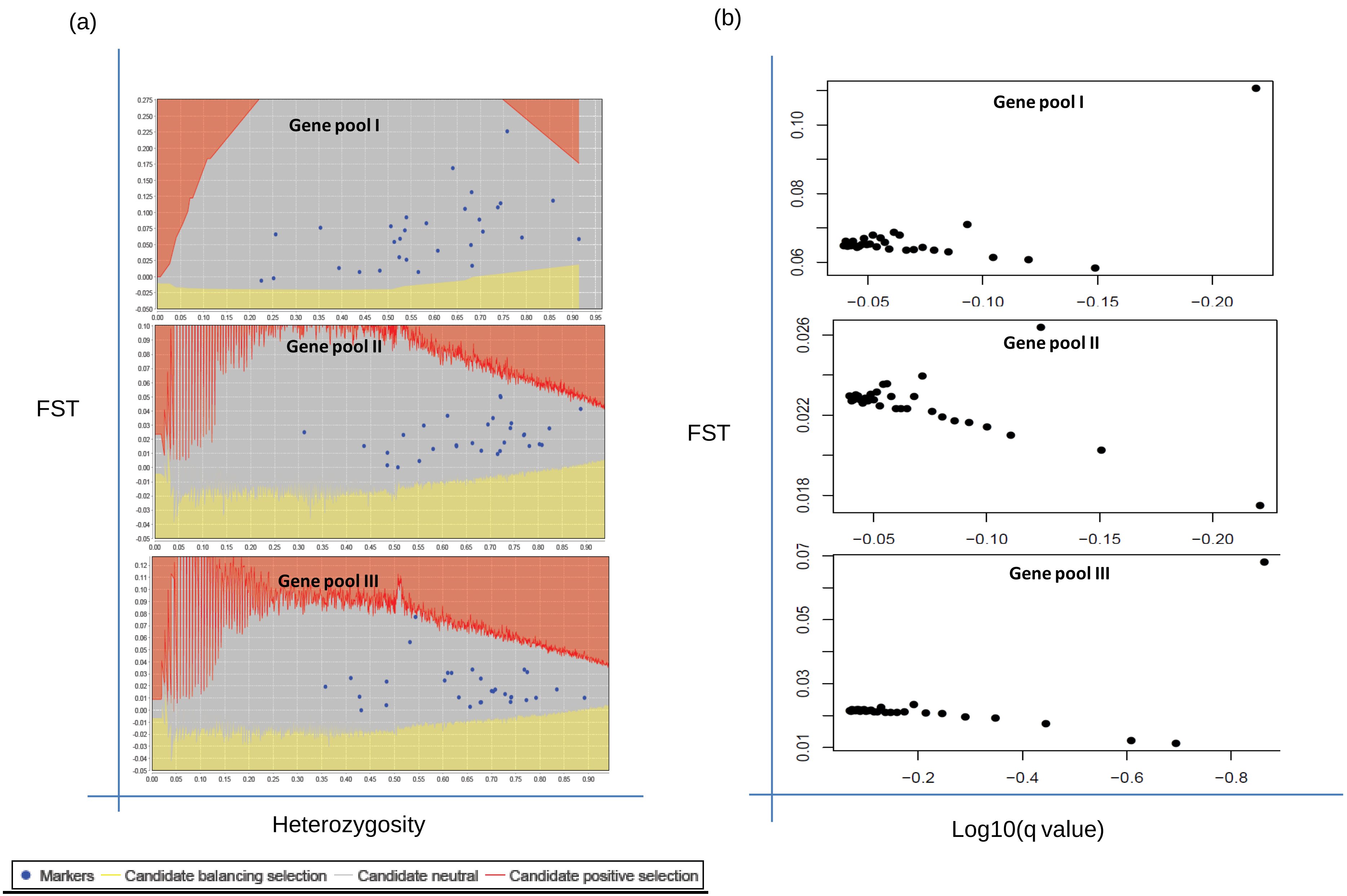

Supplementary Figure S8

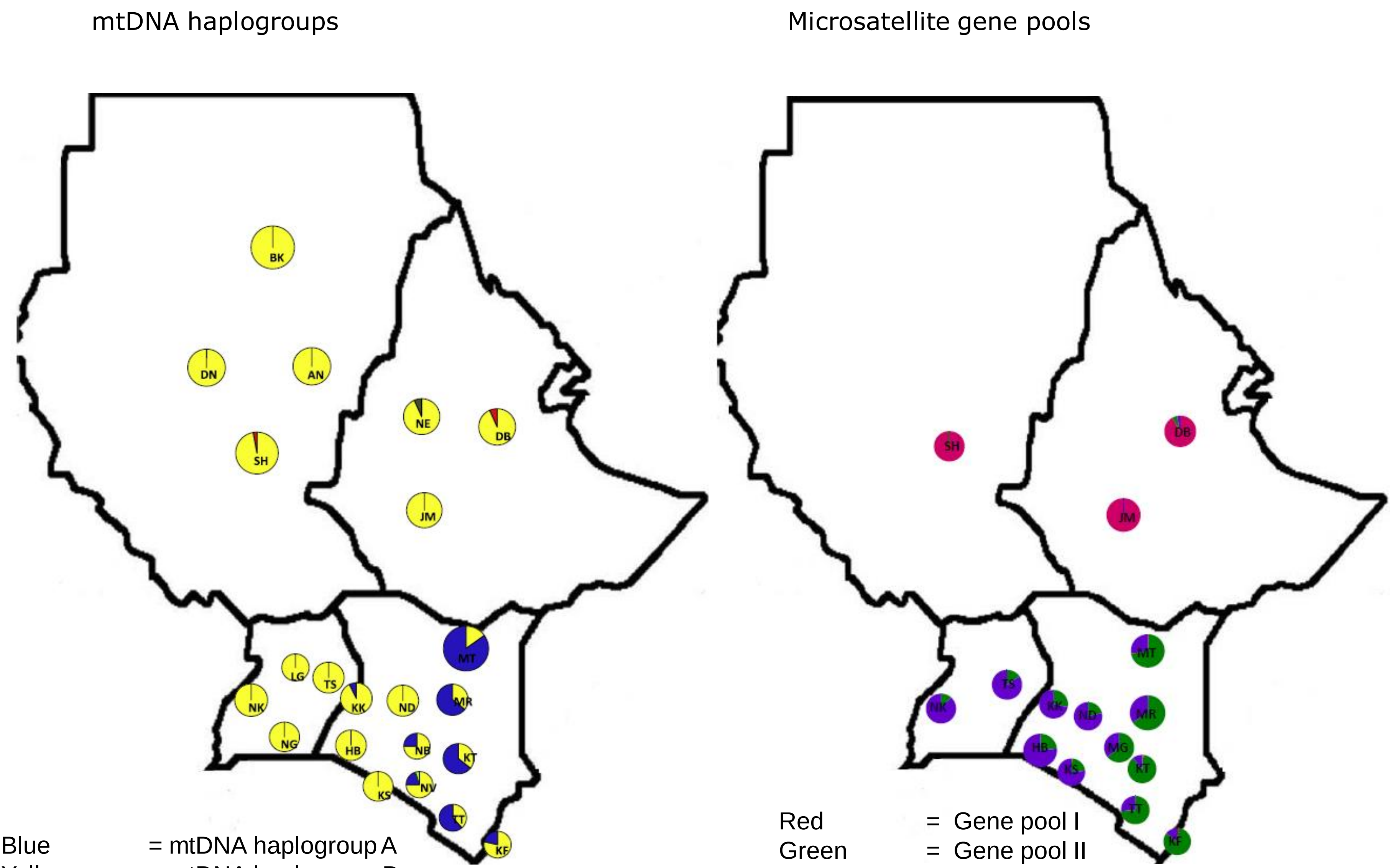

Blue = mtDNA haplogroup A
Yellow = mtDNA haplogroup D

Red = Gene pool I
Green = Gene pool II
Purple = Gene pool III
