# Supplementary material for: Reconstructing the origin and dispersal patterns of village chickens across East Africa: insights from autosomal markers
Source: Mol Ecol. 2013 Apr 24;22(10):2683–97. doi: 10.1111/mec.12294 (PMC3664419; doi:10.1111/mec.12294)
Supplement: Supplementary file 2 [file mec0022-2683-SD2.pdf]

Supplementary Information - S1

Characteristics of the alleles observed at the microsatellites loci in East African village chicken

In this study we used the 30 autosomal microsatellite markers recommended by ISAG/FAO Advisory Committee on genetic diversity in domestic animals (FAO 1998). Here we used the panel on a large sample of free-range scavenging East African village chickens. We observed a total of 285 alleles some of which had alleles outside the expected allele size range reported previously (FAO 1998, Supplementary Table S2).

From the 30 markers, five loci (MCW0216, MCW0034, MCW0037, MCW0284 and ADL0278) revealed alleles that differed by a single base pair, while 14 (MCW0216, MCW0014, MCW0183, ADL0278, MCW0104, MCW0069, MCW0034, LEI0234, MCW0016, MCW0037, LEI0094, MCW0284, LEI0192 and MCW0081), show imperfect tandem repeat allelic patterns characterized by alleles differing by a single or an uneven number of base pair alleles. To investigate whether these were not artefacts or results of scoring errors and/or double peak readings, all genotypes were double blind scored independently by five people who were conversant with microsatellites. They all returned similar results. Also, we further re-genotyped majority of the individuals with these allele patterns and each time we observed the same pattern. Another researcher working independently on Korean native chickens also observed the same pattern while another one working in The Netherlands also observed the same pattern in Ethiopian local chickens. In the published domain, similar uneven allelic patterns have also been observed in several studies (Crooijmans *et al*. 1996; Vanhala *et al*. 1998; Romanov & Weigend 2001). Using 22 out of the 30 microsatellites used in this study and genotyped in local European and Asian chicken breeds using a similar capillary sequencer (ABI 3100 Genetic Analyzer; Applied Biosystems) as the one used in our study, Berthouly *et al*. (2008) also identified unambiguously alleles with sizes differing only by one base pair in eight of the loci. Also, some of the individuals exhibiting such patterns (“atypical” alleles)

have recently been sequenced (Yin *et al*. 2011; Wei *et al*. 2011) and this has indeed confirmed the pattern to be true.

It is therefore most likely that the observed allelic patterns are an inherent characteristic of the markers in East African village chickens and they may represent either alleles that have arisen recently in these populations or have been present in the ancestral population but might have been lost in commercial breeds as a consequence of a different breeding history.

References

Berthouly C, Bed'Hom B, Tixier-Boichard M *et al*. (2008) Using molecular markers and multivariate methods to study the genetic diversity of local European and Asian chicken breeds. *Animal Genetics*, **39**, 121-129.

FAO (1998) *Secondary Guidelines for Development of National Farm Animal Genetic Resources Management Plans. Measurement of Domestic Animal Diversity (MoDAD): Recommended Microsatellite Markers*. FAO/UNEP, Rome, Italy 58pp.

Crooijmans RPMA, Groen ABF, van Kampen AJA *et al*. (1996) Microsatellite Polymorphism in Commercial Broiler and Layer Lines Estimated Using Pooled Blood Samples. *Poultry Science*, **75**, 904-909.

Romanov MN, Weigend S (2001) Analysis of genetic relationships between various populations of domestic and jungle fowl using microsatellite markers. *Poultry Science*, **80**, 1057-1063.

Vanhala T, Tuiskula-Haavisto M, Elo K *et al*. (1998) Evaluation of genetic variability and genetic distances between eight chicken lines using microsatellite markers. *Poultry Science*, **77**, 783-790.

Wei MQ, Zeng SC, Wei YM, Han J (2011) DNA sequence variations of alleles at two chicken microsatellite loci of MCW0216 and LEI0234. *Asian Journal of Animal Sciences*, **5**, 349-357.

Yin J, Zeng SC, Luo YZ, Han J (2011) Intensive DNA sequence characterization of alleles at MCW0330 and LEI0094 microsatellite loci in chicken. *Asian Journal of Animal and Veterinary Advances*, **6**, 805-813.

Supplementary Information - Tables

Supplementary Table S1. Characteristics of the ecological zones of the study area and the respective gene pools observed in each eco-zone.

Country/ Population	Eco-zone*	Description	Moisture index	Dominant Gene pool
Kenya				
East Kenya				
Kilifi	II	Coastal humid to dry sub-humid	-10	II
Taita	II	Inland humid to dry sub-humid	-10	II
Muranga	II	Humid	< -10	II
Kitui	IV	Semi arid	-30 to -40	II
Meru	III	Dry sub-humid to semi arid	-20 to -30	II
Marsabit	V	Arid	-40 to -50	II
West Kenya				
Kisii	II	Humid	< -10	III
Nandi	II	Humid	< -10	III
Homa Bay	II	Inland humid to dry sub-humid	-10	III
Kakamega	II	Humid	< -10	III
Uganda				
Teso	II	Humid	-10	III
Nkonjo	II	Humid	-10	III
Ethiopia				
Debre Berhan	II	Humid	< -10	I
Jimma	III	Dry sub-humid to semi arid	-20 to -30	I
Sudan				
Shilluk	V	Arid	-40 to -50	I

*Pratt D.J., Greenway P.J. & Gwynne M.D. (1966) A classification of East African rangeland, with an appendix on terminology. *The Journal of Applied Ecology*, **3**, 369–82.

Supplementary Table S2. Characteristics of the thirty ISAG/FAO recommended microsatellite markers used in the current study

							Allele size range	
**Locus**	**Sample size**	**No. of alleles**	**He**	**Ho**	**PIC**	**AR**	**This study**	**FAO***
MCW0123	655	10	0.673	0.650	0.643	6.796	71 - 93	76 - 100
MCW0216	655	8	0.702	0.453	0.650	5.300	134 - 151	139 - 149
MCW0034	657	13	0.805	0.731	0.780	8.662	212 - 241	212 - 246
ADL0268	657	6	0.711	0.591	0.670	5.663	100 - 114	102 - 116
MCW0016	648	14	0.761	0.657	0.725	7.204	127 - 175	162 - 206
LEI0094	653	20	0.816	0.671	0.794	10.413	236 - 287	247 - 287
MCW0165	652	5	0.575	0.319	0.495	3.198	112 - 126	114 - 118
MCW0020	652	4	0.741	0.670	0.692	4.000	177 - 183	179 - 185
MCW0222	654	4	0.662	0.564	0.597	3.992	215 - 221	220 - 226
MCW0103	654	4	0.421	0.399	0.339	2.368	260 - 268	266 - 270
MCW0295	651	10	0.702	0.619	0.664	7.092	82 - 106	88 - 106
MCW0037	652	6	0.673	0.517	0.615	5.064	148 - 153	154 - 160
LEI0234	651	25	0.898	0.751	0.889	13.805	196 - 373	216 - 364
MCW0081	657	10	0.562	0.510	0.511	5.746	109 - 140	112 - 135
MCW0014	657	9	0.344	0.139	0.326	5.269	161 - 180	164 - 182
MCW0284	635	3	0.519	0.438	0.418	2.886	236 - 245	235 - 243
MCW0183	649	12	0.755	0.704	0.725	7.251	286 - 324	296 - 326
ADL0112	653	6	0.579	0.557	0.520	4.529	120 - 130	120 - 134
MCW0104	657	17	0.671	0.560	0.643	9.114	183 - 231	190 - 234
MCW0098	657	3	0.462	0.408	0.356	2.041	255 - 259	261 - 265
MCW0111	656	10	0.654	0.613	0.619	5.620	92 - 118	96 - 120
MCW0069	654	11	0.749	0.700	0.710	7.384	152 - 174	158 - 176
MCW0206	657	13	0.742	0.673	0.700	6.581	218 - 248	221 - 249
LEI0166	655	5	0.483	0.447	0.431	3.158	338 - 354	354 - 370
ADL0278	655	13	0.764	0.592	0.723	5.993	108 - 180	114 - 126
MCW0067	657	7	0.718	0.584	0.667	4.910	170 - 182	176 - 186
LEI0192	645	20	0.811	0.553	0.786	9.956	247 - 349	244 - 370
MCW0078	654	5	0.579	0.468	0.538	4.500	134 - 142	135 - 147
MCW0248	655	4	0.424	0.388	0.367	3.420	212 - 220	205 - 225
MCW0330	654	8	0.707	0.580	0.655	5.098	254 - 286	256 - 300
**Mean**	**657**	**9.50**	**0.6554**	**0.5502**	**0.6083**	**5.90**		

* FAO (1998) *Secondary Guidelines for Development of National Farm Animal Genetic Resources Management Plans. Measurement of Domestic Animal Diversity (MoDAD): Recommended Microsatellite Markers*. FAO/UNEP, Rome, Italy 58pp.

http://dad.fao.org/cgi-bin/getblob.cgi?sid=6e227435d25a608081d877656f3f3a32,50006220

Results after removal of individuals with less than 90% of each gene pools genotype proportion

Supplementary Table S3. Number of shared alleles between the three gene pools observed in the current study

Gene Pool combination	Number of alleles	Proportion (%) out of the total number of alleles observed (274)
I and II	13	4.744
I and III	7	2.554
II and III	24	8.759
I, II and III	162	59.124

Supplementary Table S4a. Total number of gene pool specific alleles observed from the analysis of thirty microsatellite markers in 15 chicken populations

Gene Pool	Number of Alleles
I	13
II	26
III	27
Total	66

Supplementary Table S4b. Gene pool specific alleles and their frequencies

**Gene Pool**	**Locus**	**Allele**	**Frequency**
Gene pool 1	MCW0016	130	0.017699
	LEI0094	258	0.004587
	MCW0103	260	0.027273
	MCW0295	106	0.009009
	LEI0234	373	0.009009
		369	0.004505
	MCW0081	140	0.008850
	MCW0014	178	0.070796
	MCW0183	322	0.009009
	MCW0206	226	0.022124
	LEI0166	338	0.004464
	LEI0192	284	0.013274
		309	0.004425
		349	0.004425
Gene pool II	MCW0123	71	0.003165
		79	0.006329
	MCW0034	216	0.003165
		237	0.003165
		212	0.009494
		224	0.012658
	MCW0016	142	0.003165
		127	0.003165
	MCW0295	98	0.006452
	LEI0234	340	0.009677
		320	0.003226
		357	0.003226

	MCW0183	286	0.009677
		307	0.003226
	MCW0104	202	0.006329
		220	0.031646
		212	0.006329
	MCW0111	106	0.012658
	MCW0069	167	0.003205
	LEI0166	342	0.006329
	ADL0278	124	0.003165
		180	0.003165
		121	0.003165
	LEI0192	260	0.009615
		247	0.006410
		256	0.016026
		345	0.006410
Gene pool III	MCW0123	89	0.036184
	MCW0216	151	0.003289
	MCW0016	152	0.006711
		165	0.003356
		175	0.003356
	LEI0094	254	0.006579
		256	0.029605
		244	0.003289
	MCW0165	118	0.009868
	LEI0234	365	0.003289
		361	0.003289
		324	0.003289
	MCW0081	113	0.003289
		129	0.009868
	MCW0014	167	0.009868
	MCW0183	296	0.003289
	MCW0104	231	0.006579
		198	0.085526
	MCW0111	116	0.003289
	MCW0206	218	0.009868
		234	0.006579
		244	0.006579
	ADL0278	120	0.003289
	LEI0192	321	0.006667
	MCW0330	256	0.003289
		276	0.026316
		272	0.023026

Supplementary Table S5. Measures of allelic and genetic diversity for each population after excluding individuals with less than 90% of their genotype proportion from each gene pool

			**Allelic Diversity**					**Genetic Diversity**	
**Population**	**Code**	**N**	**TNA**	**MNA (SD)**	**AR (SD)**	**ENA (SD)**	**Pa**	**He (SD)**	**Ho (SD)**
Kenya									
Kilifi	KF	39	183	6.10 (2.58)	4.73 (1.53)	3.07 (1.04)	8	0.6440 (0.0238)	0.5632 (0.0145)
Taita	TT	20	154	5.13 (2.26)	4.60 (1.81)	3.16 (1.39)	3	0.6413 (0.0299)	0.5698 (0.0202)
Kitui	MG	37	167	5.57 (2.54)	4.51 (1.70)	3.12 (1.39)	2	0.6326 (0.0287)	0.5567 (0.0149)
Muranga	KT	13	137	4.57 (1.98)	4.48 (1.89)	2.98 (1.46)	0	0.6307 (0.0288)	0.5889 (0.0250)
Meru	MR	18	148	4.93 (2.00)	4.54 (1.65)	3.09 (1.18)	2	0.6485 (0.0249)	0.5664 (0.0214)
Marsabit	MT	25	148	4.93 (1.84)	4.40 (1.48)	2.92 (1.09)	2	0.6310 (0.0218)	0.5037 (0.0184)
Nandi	ND	26	166	5.53 (2.57)	4.72 (1.80)	3.26 (1.29)	4	0.6582 (0.0259)	0.5998 (0.0176)
Kisii	KS	27	169	5.63 (2.71)	4.80 (1.96)	3.31 (1.63)	0	0.6556 (0.0254)	0.5882 (0.0173)
Homa Bay	HB	26	164	5.47 (2.32)	4.62 (1.70)	3.03 (1.26)	1	0.6288 (0.0276)	0.5526 (0.0178)
Kakamega	KK	18	167	5.57 (2.78)	4.98 (2.17)	3.29 (1.40)	5	0.6682 (0.0233)	0.6089 (0.0211)
Overall	KE	249	253	8.43 (4.71)	8.40 (4.68)	3.41 (1.48)	27	0.6619 (0.0231)	0.5672 (0.0057)
Ethiopia									
Debre Berhan	DB	34	150	5.00 (2.10)	4.01 (1.51)	2.57 (1.40)	2	0.5390 (0.0344)	0.4806 (0.0157)
Jimma	JM	42	156	5.20 (2.50)	4.06 (1.56)	2.52 (1.37)	5	0.5422 (0.0302)	0.4523 (0.0141)
Overall	ET	76	179	5.97 (2.89)	5.92 (2.86)	2.70 (1.61)	7	0.5556 (0.0318)	0.4648 (0.0105)
Uganda									
Teso	TS	30	156	5.20 (2.25)	4.31 (1.60)	2.74 (1.18)	5	0.5820 (0.0310)	0.5270 (0.0167)
Nkonjo	NK	31	140	4.67 (2.04)	4.11 (1.58)	2.76 (1.14)	2	0.5875 (0.0319)	0.5209 (0.0164)
Overall	UG	61	175	5.83 (2.59)	5.80 (2.57)	2.84 (1.21)	7	0.5954 (0.0294)	0.5240 (0.0117)
Sudan									
Shilluk	SH	37	144	4.80 (2.19)	3.81 (1.38)	2.31 (0.93)	6	0.5132 (0.0310)	0.4568 (0.0151)
Across East Africa	EA	423	274	9.13 (5.41)	4.84 (1.81)	3.38 (1.63)	47	0.6528 (0.0244)	0.5329 (0.0044)
